# Prevalence of infant sneezing without colds and prediction of childhood allergy diseases in a prospective cohort study

**DOI:** 10.18632/oncotarget.22338

**Published:** 2017-11-07

**Authors:** Kuender D. Yang, Chih-Chiang Wu, Ming-Tsung Lee, Chia-Yu Ou, Jen-Chieh Chang, Chih-Lu Wang, Hau Chuang, Ho-Chang Kuo, Chie-Pein Chen, Te-Yao Hsu

**Affiliations:** ^1^ Department of Pediatrics, Mackay Memorial Hospital, Taipei, Taiwan; ^2^ Institute of Biomedical Sciences, Mackay Medical College, New Taipei City, Taiwan; ^3^ Institute of Clinical Medicine, National Yang-Ming University, Taipei, Taiwan; ^4^ Research Assistance Center, Show Chwan Memorial Hospital, Changhua, Taiwan; ^5^ Department of Pediatrics, Po-Jen Hospital, Kaohsiung, Taiwan; ^6^ Department of Pediatrics and Medical Research, Kaohsiung Chang Gung Memorial Hospital and Chang Gung University College of Medicine, Kaohsiung, Taiwan; ^7^ Department of Obstetrics and Gynecology, Mackay Memorial Hospital, Mackay Medical College, Taiwan; ^8^ Department of Obstetrics and Gynecology, Kaohsiung Chang Gung Memorial Hospital, Taiwan and Chang Gung University College of Medicine, Kaohsiung, Taiwan

**Keywords:** asthma, allergic rhinitis, atopic dermatitis, birth cohort, infant sneezing without colds

## Abstract

**Background:**

Allergy sensitization may begin during the perinatal period, but predicting allergic diseases in infancy remains difficult. This study attempted to identify early predictors of childhood allergy diseases in a prospective cohort study.

**Materials and Methods:**

In a prospective birth cohort study at southern Taiwan locating in a subtropical region, questionnaire surveys of sneezing or cough without colds at 6 and 18 months of age were recorded, and the correlation with allergy diseases was assessed at 3 and 6 years of age.

**Results:**

A total of 1812 pregnant women and 1848 newborn infants were prenatally enrolled, and 1543, 1344, 1236, and 756 children completed the follow-up at ages 6 months, 18 months, 3 years and 6 years, respectively. The prevalence of infant sneezing without colds at 6 and 18 months of age was 30.3% and 19.2%, respectively. The prevalence of infant cough without colds at 6 and 18 months of age was 10.6% and 5.7%, respectively. Infant sneezing without colds at 18 months of age was significantly correlated with atopic dermatitis, allergic rhinitis and asthma at 6 years of age. Infant cough without colds at 18 months of age significantly predicted asthma but not atopic dermatitis or allergic rhinitis at 6 years of age.

**Conclusions:**

Infant sneezing without colds predicted all allergy diseases at 6 years of age in a subtropical country. This highlights a potential non-invasive clue in a subtropical region for the early prediction, treatment and prevention of childhood allergy diseases in infancy.

## INTRODUCTION

The prevalence of childhood allergy diseases has increased worldwide in recent decades [1]. Many studies have shown that allergy diseases are complex diseases related to gene-environment interactions, which makes the early prediction and prevention of allergy diseases difficult. Traditionally, parental atopy history has been the most important predictor of the development of allergy diseases in offspring [2, 3]. However, recent evidence suggests that early life infections [4] and a green environment (forest and agricultural land) within 5 kilometers [5] have different impacts on the development of childhood asthma. Children born by cesarean section have an increased likelihood of asthma at 36 months of age, and the association is stronger among children of nonatopic mothers [6]. Elevated total immunoglobulin E (IgE), frequent respiratory infections, and parenting difficulties in the first year of life were associated with asthma at 3 and 6 years of age [7].

To observe and identify risk factors for early prediction and prevention of childhood allergic diseases, we conducted a birth cohort study in Kaohsiung, Taiwan, as reported previously [8–11]. We found that elevated maternal but not paternal total IgE levels correlated with elevated infant IgE levels and infant atopy [8]. Atopic disease in a mother increases the risk of atopic eczema in her child but is a poor predictor of atopic eczema [8]. Breastfeeding, Cesarean section, use of curtains and/or air filters affect development of atopic dermatitis (AD), allergic rhinitis (AR) and asthma (AS) in offspring of non-atopic parents [12]. These results suggest that perinatal factors in addition to parental inheritance have an impact on the development of childhood allergic diseases and that it is important to identify an early infant predictor to facilitate the prediction and prevention of childhood allergy diseases.

Recently, the hygiene hypothesis has suggested that infants with less exposure to microorganisms in the environment are more susceptible to the development of allergic diseases [13, 14]. By contrast, Nja et al. [15] indicated that a history of lower respiratory infections in infancy was a risk factor for asthma in school-age children. Moreover, children with asthma or other atopic diseases are more susceptible to infections [16]. Whether increased infections in infancy cause asthma or infant occult asthma or allergic sensitization causes increased infections remains controversial. Our previous analysis demonstrated that the gene-environment interaction on allergic sensitization begins in the perinatal stage [9, 10]. Infant wheezing combined with other risk features was recently used to predict persistent asthma before 3 years of age [17–19]. However, wheezing is related to transient or non-atopic wheezing in two thirds of infants with wheezing episodes [20, 21]. Our previous study also showed that frequent infant wheezing was not correlated with allergy sensitization but was correlated with Clara cell protein 10 (CC10) expression [22]. Based on these findings, we attempted to investigate whether early infant sneezing or infant cough without colds was correlated with perinatal conditions of parents or infants and thus could be used to predict childhood allergy diseases. Accordingly, this study acquired information on early (6 months) and late (18 months) infant sneezing or cough without colds to predict the development of allergic rhinitis (AR), atopic dermatitis (AD) and asthma (AS) at 3 and 6 years of age.

## RESULTS

### Demographic data of parents and their offspring in the birth cohort

**Figure 1:**
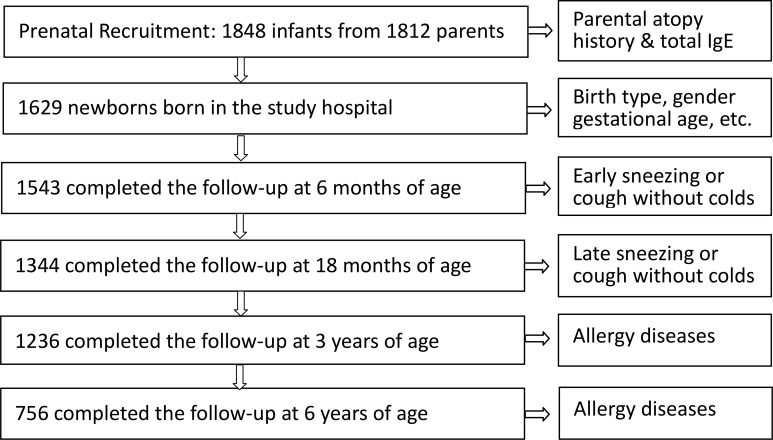
Summary of the birth cohort study A total of 1848 infants from 1812 pairs of parents were prenatally recruited. Of the enrolled newborns, 1543, 1344, 1236 and 756 completed follow-up at 6 months, 18 months, 3 years and 6 years of age, respectively.

In this study, 1848 newborns were prenatally recruited, of which 1543, 1344, 1236, and 756 children completed the follow-up at the age of 6 months, 18 months, 3 years and 6 years, respectively (Figure 1). In total, 1000, 849, 852, and 737 children underwent blood tests to measure total IgE and specific IgE levels with parental permission at the follow-up visits at 6 months, 18 months, 3 years and 6 years of age, respectively. The cohort population was 53.3% male, 6.5% preterm (< 37 weeks gestational age), 28.2% Cesarean section, 54.5% maternal allergy history, 44.4% paternal allergy history, 26.5% maternal allergy disease, and 36.7% paternal allergy disease. The mothers in this cohort population reported greater allergy history but less definite allergy disease as defined by allergic disease history with elevated IgE levels ≥ 100 kU/l. There were no significant differences in the demographic data of the participants who did or did not complete the 6-year follow-up, as described elsewhere [11].

### Prevalence of infant sneezing or cough without colds

We collected data on early and late infant sneezing or cough without colds at 6 and 18 months of age, respectively. The prevalence of early infant sneezing without colds was 30.3%, including occasional episodes in 25.8% and frequent episodes in 4.5%. The prevalence of early infant cough without colds was 10.6%, including occasional episodes in 9.7% and frequent episodes in 0.9% (Table 1A). The prevalence of late infant sneezing without colds was 19.2%, including occasional episodes in 16.4% and frequent episodes in 2.8%. The prevalence of late infant cough without colds was 5.7%, including occasional episodes in 4.8% and frequent episodes in 0.9% (Table 1B).

**Table 1A:** Prevalence of early infant sneezing or cough without colds at 6 months of age

Symptoms	Frequency	Number	Percentage
Sneezing	No symptoms	1076	69.70%
	Occasional	398	25.80%
	Frequent	69	4.50%
Cough	No symptoms	1380	89.40%
	Occasional	149	9.70%
	Frequent	14	0.90%

**Table 1B:** Prevalence of early infant sneezing or cough without colds at 18 months of age

Symptoms	Frequency	Number	Percentage
Sneezing	No symptoms	1087	80.90%
	Occasional	220	16.40%
	Frequent	37	2.80%
Cough	No symptoms	1267	94.30%
	Occasional	65	4.80%
	Frequent	12	0.90%

Notes: Occasional was defined as < 3 days per week, and frequent was defined as ≥ 3 days per week in the past 6 months.

### Late but not early infant sneezing is associated with allergy diseases at 3 or 6 years of age

Analyses were next performed to assess whether early sneezing at 6 months or late sneezing at 18 months was a predictor of allergic diseases at 3 and 6 years of age by Chi-square for trend analysis in the comparison among the 3 subgroups (no symptom, occasional or frequent). Early infant sneezing at 6 months of age was not significantly associated with atopic dermatitis, allergic rhinitis and asthma at 3 and 6 years of age (Table 2A). Late infant sneezing at 18 months of age was significantly associated with atopic dermatitis (*p =* 0.002) and allergic rhinitis (< 0.001) but not asthma (*p =* 0.843) at 3 years of age (Table 2B). Remarkably, late infant sneezing without colds was significantly associated with atopic dermatitis (0.018), allergic rhinitis (0.012) and asthma (0.001) at 6 years of age (Table 2B).

**Table 2:** Infant sneezing or cough without colds associated with childhood allergy diseases

A) Early infant sneezing without colds at 6 months of age					
	No Symptoms		Occasional	Frequent	*p*
Allergy diseases at 3 years of age					
	(792)	(324)		(57)	
Atopic dermatitis	9.8% (78)	13.9% (45)		10.5% (6)	0.146
Allergic rhinitis	13.0% (103)	10.5% (34)		8.8% (5)	0.370
Asthma	5.7% (45)	5.9% (19)		12.3% (7)	0.129
Allergy diseases at 6 years of age					
	(506)	(184)		(34)	
Atopic dermatitis	13.4% (68)	15.8% (29)		17.6% (6)	0.626
Allergic rhinitis	57.1% (289)	60.3% (111)		61.8% (21)	0.683
Asthma	24.9% (126)	24.5% (45)		32.4% (11)	0.606
**B) Late infant sneezing without colds at 18 months of age**					
Allergy diseases at 3 years of age					
	(887)	(192)		(32)	
Atopic dermatitis	9.8% (86)	17.2% (33)		21.9% (7)	0.002
Allergic rhinitis	10.3% (90)	18.2% (35)		28.1% (9)	<0.001
Asthma	5.7% (50)	4.7% (9)		6.3% (2)	0.843
Allergy diseases at 6 years of age					
	(561)	(114)		(25)	
Atopic dermatitis	12.5% (70)	14.9% (17)		32% (8)	0.018
Allergic rhinitis	55.6% (312)	67.5% (77)		76% (19)	0.012
Asthma	23.0% (129)	28.9% (33)		56.0% (14)	0.001
**C) Early infant cough without colds at 6 months of age**					
Allergy diseases at 3 years of age					
	(1033)		(127)	(13)	
Atopic dermatitis	10.0% (103)		18.1% (23)	23.1% (3)	0.008
Allergic rhinitis	12.2% (126)		11.8% (15)	7.7% (1)	0.880
Asthma	5.9% (61)		7.9% (10)	0.0% (0)	0.445
Allergy diseases at 6 years of age					
	(646)		(71)	(7)	
Atopic dermatitis	14.4% (93)		14.1% (10)	0.0% (0)	0.555
Allergic rhinitis	57.0% (368)		67.6% (48)	71.4% (5)	0.175
Asthma	24.0% (155)		33.8% (24)	42.9% (3)	0.108
**D) Late infant cough without colds at 18 months of age**					
Allergy diseases at 3 years of age					
	(1040)		(51)	(10)	
Atopic dermatitis	10.7% (111)		25.5% (13)	20.0% (2)	0.004
Allergic rhinitis	12.1% (126)		11.8% (6)	20.0% (2)	0.747
Asthma	5.4% (56)		9.8% (5)	0.0% (0)	0.300
Allergy diseases at 6 years of age					
	(658)		(34)	(8)	
Atopic dermatitis	13.4% (88)		17.6% (6)	12.5% (1)	0.774
Allergic rhinitis	58.2% (383)		64.7% (22)	37.5% (3)	0.368
Asthma	24.0% (158)		44.1% (15)	37.5% (3)	0.022

Notes: *P* values are calculated from Chi-square for trend analysis in the comparison among the 3 subgroups (no symptom, occasional or frequent).

We also assessed whether early or late infant cough without colds at 6 months or 18 months of age predicted allergic diseases at 3 and 6 years of age. Early infant cough without colds was significantly associated with atopic dermatitis (*p =* 0.008) but not allergic rhinitis or asthma at 3 years of age; late infant cough without colds was significantly associated with asthma but not AR or AD at 6 years of age (*p =* 0.022) (Table 2B).

### Late infant sneezing without colds is correlated with parental and childhood aeroallergen sensitization

To investigate the perinatal factors and childhood allergen sensitization associated with late infant sneezing or cough without colds at 18 months, we performed a Chi-square analysis for the trend to identify predictors. Late infant sneezing without colds was significantly associated with maternal allergy disease (*p =* 0.020), paternal allergy disease (*p =* 0.028) and childhood aeroallergen sensitization (*p =* 0.023) but not frequent URIs (≥ 3 times) (*p =* 0.051) (Table 3A). By contrast, late infant cough without colds at 18 months of age was significantly associated with frequent URIs (≥ 3 times) (*p =* 0.016) and childhood aeroallergen sensitization (*p =* 0.011) but not maternal or paternal allergy disease (Table 3B). Tobacco smoke exposure (TSE) was not significantly associated with infant sneezing without colds or infant cough without colds. The high rates (62.4–90.9%) of frequent URIs (≥ 3 times) were reported in different groups of infant sneezing or cough without colds, suggesting early infant allergic symptoms with cough or sneezing could be potentially recalled as common colds by parents.

**Table 3A:** Infant sneezing without colds at 18 months of age associated with perinatal and childhood conditions

Condition	no sneezing	occasional	frequent	*p*
Male gender	53.3%	54.1%	59.5%	.527
Preterm (< 37 weeks)	6.0%	9.5%	5.4%	.182
Cesarean section	27.0%	31.6%	27.8%	.306
Prenatal TSE	22.6%	25.2%	28.6%	.139
Frequent URIs^*^	62.4%	69.3%	75.7%	.051
Ma. allergy disease	26.6%	35.2%	32.4%	.020
Pa. allergy disease	36.1%	41.0%	51.4%	.028
Ma. allergy history	52.9%	59.5%	56.8%	.108
Pa. allergy history	43.0%	49.5%	48.6%	.083
Aeroallergen IgE	10.4%	14.6%	22.2%	.023
Food allergen IgE	22.0%	25.8%	25.9%	.308

**Table 3B:** Infant cough without colds at 18 months of age associated with perinatal and childhood conditions

Condition	no sneezing	occasional	frequent	*p*
Male gender	53.4%	53.8%	66.7%	.520
Preterm (< 37 weeks)	6.6%	4.6%	16.7%	.647
Cesarean section	27.7%	26.2%	40.0%	.731
Prenatal TSE	23.4%	20.0%	20.0%	.806
Frequent URIs^*^	63.0%	76.6%	90.9%	.016
Ma. Allergy disease	28.2%	29.7%	25.0%	.970
Pa. Allergy disease	36.9%	46.0%	33.3%	.362
Ma. allergy history	53.8%	56.9%	75.0%	.184
Pa. allergy history	44.1%	41.5%	66.7%	.479
Aeroallergen IgE	10.7%	23.3%	22.2%	.011
Food allergen IgE	22.6%	25.6%	33.3%	.397

Notes: TSE, tobacco smoke exposure; Ma., Maternal; Pa., paternal. ^*^ Frequent URIs was defined as > 2 URIs in the past 6 months. *P* values were calculated by Chi-square for trend analysis.

### Prediction of childhood allergic diseases by late infant sneezing or cough without colds

We next used a 2 × 3 table to assess the relative risk (RR) of the allergy diseases of AD, AR and AS at 3 and 6 years of age based on late infant sneezing or cough without colds at 18 months. Sneezing described as “occasional” was associated with a significantly higher predictive risk of AD (*p =* 0.007, RR = 1.872) and AR (*p =* 0.005, RR = 1.893) but not AS at 3 years of age. Similarly, sneezing described as “frequent” was associated with a significantly higher predictive risk of AD (*p =* 0.032, R R= 2.610) and AR (*p =* 0.003, RR = 3.410) but not AS at 3 years of age (Table 4, upper panel). By contrast, only sneezing described as “frequent” was associated with a significantly higher predictive risk of AD (*p =* 0.007, RR = 3.367) and AS (*p =* 0.002, RR=3.815) but not AR (*p =* 0.090, RR = 2.263, 95% CI 0.880–5.821) at 6 years of age (Table 4, lower panel). Late infant cough described as “occasional” was associated with a significant predictive risk of AD (*p =* 0.002, RR=2.904) but not AR or AS at 3 years of age (Table 5, upper panel). By contrast, late infant cough described as “occasional” was associated with a significant predictive risk of AS (*p =* 0.026, RR=2.306) but not AD or AR at 6 years of age (Table 5, lower panel). The results of these analyses suggest that infant sneezing without colds described as “frequent” is more predictive of different allergy diseases at 3 and 6 years of age, whereas infant cough without colds described as “occasional” is predictive of AD at 3 years of age and AS at 6 years of age.

**Table 4:** Relative risk (RR) of allergy diseases predicted by infant sneezing without colds

Sneezing	RR to AD at 3 years of age	*p*	RR to AR at 3 years of age	*p*	RR to AS at 3 years of age	*p*
No symptoms	1	−	1	−	1	−
Occasional	1.872 (1.190–2.946)	0.007	1.893 (1.216−2.948)	0.005	0.750 (0.343−1.640)	0.471
Frequent	2.610 (1.087–6.264	0.032	3.410 (1.512−7.688)	0.003	1.013 (0.228−4.500)	0.987
Sneezing	RR to AD at 6 years of age	*p*	RR to AR at 6 years of age	*p*	RR to AS at 6 years of age	*p*
No symptoms	1		1		1	
Occasional	1.164 (0.640–2.117)	0.618	1.513 (0.975−2.348)	0.065	1.184 (0.735−1.906)	0.487
Frequent	3.367 (1.384–8.191)	0.007	2.263 (0.880−5.821)	0.090	3.815 (1.665−8.744)	0.002

Notes: AD, atopic dermatitis; AR, allergic rhinitis; AS, asthma.

**Table 5:** Relative risk (RR) of allergy diseases predicted by infant cough without colds

Cough	RR to AD at 3 years of age	*p*	RR to AR at 3 years of age	*p*	RR to AS at 3 years of age	*p*
No symptoms	1		1		1	
Occasional	2.904 (1.487–5.673)	0.002	0.989 (0.410–2.385)	0.981	1.682 (0.566–5.003)	0.349
Frequent	2.439 (0.495–12.014)	0.273	1.965 (0.400–9.645)	0.405	-	0.999
Cough	RR to AD at 6 years old	*p*	RR to AR at 6 years old	*p*	RR to AS at 6 years old	*p*
No symptoms	1		1		1	
Occasional	1.478 (0.587–3.725)	0.407	1.234 (0.580–2.626)	0.585	2.306 (1.105–4.809)	0.026
Frequent	1.128 (0.133–9.604)	0.912	0.369 (0.085–1.595)	0.369	1.535 (0.352–6.699)	0.568

Notes: AD, atopic dermatitis; AR, allergic rhinitis; AS, asthma.

## DISCUSSION

This study revealed that infant sneezing without colds with morning or night symptoms at 18 months of age significantly predicted all allergy diseases at 6 years of age. Infant cough without colds with morning or night symptoms at 18 months of age only predicted childhood asthma but not atopic dermatitis or allergic rhinitis at 6 years of age. These results highlight the potential for early prediction of allergy diseases during infancy based on sneezing or cough without colds.

It remains difficult to predict or diagnose allergic rhinitis and asthma in infants or toddlers. Historically, infant and childhood allergy diseases have been predicted based on parental atopic disease [2, 3]. However, allergy diseases are complex diseases that cannot be accurately predicted by inheritance. Many studies have recognized that infant wheezing in combination with parental atopy (asthma or eczema) and infant risk features (eosinophilia, eczema, allergen sensitization) can predict later asthma before 3 years of age [17–20]. Recently, Pescatore et al. [23] developed a simple asthma prediction tool with 10 non-invasive symptoms and signs for preschool children who wheeze or cough and observed that sex, age, wheezing without colds, wheezing frequency, activity disturbance, shortness of breath, exercise-related and aeroallergen-related wheezing/coughing, eczema, and parental history of asthma/bronchitis predicted school children with asthma in a birth cohort in the United Kingdom. This tool was shown to be useful for the prediction of childhood asthma in high-risk toddlers in a subsequent German birth cohort study [24]. This novel 10-item asthma prediction tool is non-invasive and simple but too long to remember and record in clinical practice. Moreover, the tool emphasizes 4 types of wheezing features (wheezing frequency, exercise-related wheezing, wheezing without colds and aeroallergen-related wheezing) in the 10 non-invasive symptoms [23], which may be redundant.

In fact, infant wheezing can be classified into 3 populations: transient, nonatopic and allergy-related wheezing. Most wheezing during the first 3 years of life is related to transient wheezing and non-atopic wheezing, and allergy-related infant wheezing occurs in less than one-third of the population exhibiting wheezing [17–19, 22]. These observations were replicated in the Copenhagen (COPSAC2000) birth cohort, which revealed that a global assessment of significant lung symptoms in the first 3 years of life is a better predictor of asthma than an assessment of wheezing [21]. In the present study in a subtropical country where house dust mite-mediated allergic sensitization is dominantly prevalent, we have demonstrated that infant sneezing without colds with morning or night symptoms significantly predicted all allergy diseases at 6 years of age, whereas infant cough without colds only significantly predicted asthma and not atopic dermatitis or allergic rhinitis.

Coughing and sneezing are common cold symptoms and thus have not been used as early predictors of allergic diseases in questionnaires in many birth cohort studies. Although we included cough or sneezing without colds at morning or night in the proposed questionnaire, we observed that infant sneezing without colds at 18 months of age was a better predictor of allergy diseases at 6 years of age than infant cough without colds. This result may have been obtained because morning or night cough without colds is more likely to be a confounder of common colds, whereas morning or night sneezing without colds is more likely to be related to allergic symptoms. This possibility is further supported by our data indicating that infant sneezing without colds was significantly associated with parental allergy disease and the child’s aeroallergen sensitization; infant cough without colds was significantly associated with URIs but not parental allergy disease (Table 3).

The prevalence of infant sneezing without colds at 6 months of age was 30.3% and decreased to 19.2% at 18 months of age, whereas infant sneezing without colds at 18 months of age predicted allergic diseases better than that at 6 months of age, suggesting that infant sneezing without colds at younger than 6 months of age are more related to nonallergic trigger(s) and those at 18 months of age are more related to allergic trigger(s). Few studies have demonstrated that infant sneezing is associated with or predicts childhood allergy diseases. In this study, we attempted to differentiate whether the frequency of sneezing or cough without colds predicted allergy diseases at 6 years of age. We observed that infant sneezing reported as “frequent” at 18 months of age significantly predicted atopic dermatitis and allergic rhinitis at 3 years of age, whereas sneezing reported as “frequent” was the best predictor of asthma at 6 years of age. By contrast, the size 13 subjects included in the “Frequent” infant cough without colds provided not enough power for statistical analysis, however, the sizes between 34 and 127 subjects in the group of “Occasional” cough without colds is good enough for analyses on the prediction of childhood asthma at 6 years of age. Moreover, the populations of AD, AR and AS might be overlapped each other, we, however, did not analyze the multiplex interactions (sneezing and/or cough in frequent, occasional and no symptom vs. AD, AR and AS) because the sizes in subgroups are not qualified for the analyses.

The prevalence of children with allergic rhinitis (AR) at 6 years of age while defined by symptoms of easy sneezing and/or itching eyes for longer than 2 weeks in the past six months and had been diagnosed with AR by a physician was 55.6, 67.5 and 76.0%, respectively, in infants with no sneezing, occasional sneezing and frequent sneezing at 18 months of age. The prevalence is higher than those (30.8-50.7%) defined by physician-diagnosed rhinitis with detection of specific aeroallergen IgE in our previous study [12]. We did not use the latter definition in this study because we measured specific IgE only in 2 common aeroallergens (house dust mite and cockroach).

The strength of this study is the longitudinal birth cohort study with a large sample size. We were able to include parental and infant factors for the prediction of allergic diseases in children at 3 and 6 years of age. It was also effective to define sneezing or cough without colds as morning and/or night symptoms in the questionnaire at 6 months and 18 months of age. The responses of parents with respect to sneezing without colds may be inconsistent depending on the seasons in which they returned to the outpatient clinics for follow-up. Fortunately, this birth cohort was performed in Taiwan, a subtropical island, where perennial AR and AS are predominantly associated (> 90%) with house dust mite and/or German cockroach sensitization and pollen-related AR and AS are rarely observed [11, 22, 25, 26]. Thus, the prediction of AR and AS by infant sneezing may be limited to regions in which house dust mite-mediated perennial AR and AS are prevalent. Other limitations of this study include the following: 1) the relative high retention rates at newborn, 18 months and 3 years of age (> 75%) but lower retention rate at 6 years of age may have affected the power of the prediction of allergic diseases in children at 6 years of age; 2) Although infant cough without colds (no symptom, occasional or frequent) in the Chi-square for trend analysis revealed a significant trend on prediction of AS, the sizes of populations in AD, AR or AS for the group ≥ 3 days per week were less than 13 (between 7 and 13), suggesting the statistical power is not qualified for the inference; 3) using questionnaires to collect allergic symptoms by parents may have a recall bias, although we made a group consensus to collect the data by differentiating common colds with a course of 5 to 7 days of continual daily symptoms from infant sneezing or cough without colds at morning and/or night occurrence more than 2 weeks in the last 6 months. In future studies, we must refine a quantitative scale in an App (application software) for the collection of data on infant sneezing or cough without colds such as the “Mymee software” at https://qz.com/507727/a-man-who-recorded-his-every-sneeze-for-five-years-might-have-a-fix-for-your-pollen-allergy/ built to support iterative health monitor for each individual to validate the early symptoms of allergy sensitization and allergy diseases.

In summary, we defined and differentiated common colds as a course of 5 to 7 days of continual daily cough or sneezing symptoms from infant sneezing or cough without colds as morning/night sneezing or cough for more than 2 weeks in the past 6 months in a subtropical country to present a better predictor of childhood allergic diseases in infancy. This study highlights the potential for early prediction of allergy diseases during infancy based on non-invasive symptoms. We demonstrated that infant sneezing without colds at 18 months of age could predict all allergic diseases at 6 years of age, whereas infant cough without colds could predict only AS because of the lower rate of infant cough without colds. Moreover, we found that allergic rhinitis appears to develop earlier than asthma because the prevalence of infant sneezing at 18 months of age was much higher than that of cough without colds and the prevalence of AR at 3 years of age was also much higher than that of AS. Based on this birth cohort study in a subtropical region where perennial allergy diseases are prevalent, the march of allergies from AD, AS to AR might be modified to AD, AR to AS. Further studies are needed to determine whether a better design with keeping track of infant sneezing or cough without colds in an App at weekly basis may predict childhood AR and AS in the countries where seasonal and/or perennial allergy diseases are prevalent.

## MATERIALS AND METHODS

### Study design and subjects

This study is part of a longitudinal birth cohort study conducted in Kaohsiung, Taiwan, as reported previously [8–11]. A total of 1812 pregnant women and 1848 newborn infants were prenatally recruited after informed consent was obtained. A research nurse was trained to explain the purpose of this study to eligible pregnant women when they visited obstetric clinics. Upon recruitment, information on parental allergic history such as asthma, allergic rhinitis, and/or atopic dermatitis was acquired from the questionnaire, and parental blood tests to determine IgE levels were performed in the second or third trimester upon consent. At delivery, the type of delivery, prematurity defined as a gestational age of < 37 weeks, and gender were recorded. Infants and children were followed at 6 months, 18 months, 3 years and 6 years of age. Blood tests for total IgE and specific IgE levels were also performed at the clinical follow-ups. The study protocol was reviewed and approved by the Institutional Review Board committee of Chang Gung Memorial Hospital.

### Questionnaires for collecting information on early infant sneezing or cough without colds

Infants at 6 and 18 months of age were followed in pediatric clinics, where questionnaires were administered to their parents to collect information on frequency (0, 1–2 or > 2 times in the past 6 months) of URIs, frequency (no symptoms, occasional (< 3 days a week) or frequent (≥ 3 days a week)) of sneezing without colds with morning and/or night onset, and frequency (no symptoms, occasional (< 3 days a week) or frequent (≥ 3 days a week)) of cough without colds with morning and/or night onset.

### Questionnaires for clinical allergy diseases at 3 and 6 years of age

Children at 3 and 6 years of age were assessed for allergy diseases in pediatric clinics, and parents were asked 1) whether their child had AD (chronic or relapsing eczema) lasting longer than 2 weeks in the past six months and had been diagnosed with AD by a physician; 2) whether their child had AR symptoms of easy sneezing and/or itching eyes for longer than 2 weeks in the past six months and had been diagnosed with AR by a physician; and 3) whether their child had more than three asthmatic wheezing episodes and had been diagnosed with AS by a physician. The content of the questionnaire reports was verified by an allergist at follow-up in the pediatric clinics.

### Detection of total IgE and specific allergen IgE levels

Sera were collected from parents and their offspring, centrifuged at 3000 rpm (1500 g) for 15 minutes, and stored at −80°C until analysis. Serum total IgE levels and specific IgE antibodies in the blood of children at 3 and 6 years of age were determined by a full-range total IgE detection system to measure total IgE and specific IgE levels to egg white (f1), cow’s milk (f2), peanut (f13), shrimp (f24), house dust mite (d1), and German cockroach (i6) (Pharmacia & Upjohn Diagnostics AB, Uppsala, Sweden). The specific IgE profiles to these 6 common allergens were assessed based on a pilot study of 100 random samples to identify common allergens, which were defined by greater than 5% allergen sensitization (specific IgE ≥ 0.35 KU/l) in the population studied [8, 11]. Aeroallergen sensitization was defined by specific IgE to aeroallergen (house dust mite or German cockroach) ≥ 0.35 kU/L and food allergen sensitization was defined by specific IgE to egg white, cow’s milk, peanut or shrimp ≥ 0.70 kU/L. Allergy disease of the parents was defined by the presence of allergy disease history together with a total IgE level ≥ 100 kU/L [8, 11].

### Data analysis and statistics

Data were coded in an Excel file with a series of labels for demographic, clinical and laboratory data but not participants’ names. Allergic symptoms and allergy diseases were presented as percentages and subjected to Chi-square for trend analysis in the comparison among the 3 subgroups (no symptom, occasional or frequent). Relative risks with 95% CI values were assessed by a Poisson regression model and used to predict the development of childhood allergy diseases based on infant sneezing or cough without colds. The populations of AD, AR and AS might be overlapped each other, we should have analyzed effects of infant sneezing or cough without colds on the comorbidities among AD, AR and/or AS. We did not analyze the multiplex interactions (sneezing and/or cough in frequent, occasional and no symptom vs. AD, AR and AS) because the sizes in subgroups are not qualified for the analyses. For all statistical computations, SPSS for Windows, version 17.0 (Chicago, IL, USA), was used. A *p* value of ≤ 0.05 was considered statistically significant.
